# Biomarkers to optimize PSMA-targeted radioligand therapy for metastatic castration-resistant prostate cancer

**DOI:** 10.3389/fonc.2025.1583168

**Published:** 2025-08-20

**Authors:** Himisha Beltran, Jeremie Calais, Louise Emmett, Phillip H. Kuo, Christopher J. Logothetis

**Affiliations:** ^1^ Department of Medical Oncology, Dana-Farber Cancer Institute, Boston, MA, United States; ^2^ Ahmanson Translational Theranostics Division, Department of Molecular and Medical Pharmacology, David Geffen School of Medicine at UCLA, University of California, Los Angeles, Los Angeles, CA, United States; ^3^ Department of Theranostics and Nuclear Medicine, St Vincent’s Hospital Sydney, St Vincent’s Clinical School, University of New South Wales, Sydney, NSW, Australia; ^4^ Department of Radiology, City of Hope National Medical Center, Duarte, CA, United States; ^5^ Department of Genitourinary Medical Oncology, MD Anderson Cancer Center, Houston, TX, United States

**Keywords:** metastatic castration-resistant prostate cancer, prostate-specific membrane antigen, radioligand therapy, [^177^Lu]Lu-PSMA-617, biomarkers

## Abstract

Although the recently approved prostate-specific membrane antigen (PSMA)-targeted radioligand therapy (RLT) [^177^Lu]Lu-PSMA-617 has improved outcomes for patients with metastatic castration-resistant prostate cancer (mCRPC), not all patients respond optimally to this treatment; even measuring response accurately can be difficult. Moreover, there is currently a lack of validated prognostic and predictive biomarkers for [^177^Lu]Lu-PSMA-617 treatment in this patient population. There is, therefore, a growing need to identify biomarkers to help optimize patient selection for [^177^Lu]Lu-PSMA-617 and guide therapy decision-making. This review explores the landscape of emerging clinical, molecular, and imaging biomarkers, and their potential utility as prognostic and/or predictive biomarkers in the context of [^177^Lu]Lu-PSMA-617 treatment for patients with mCRPC.

## 1 Introduction

In the United States, prostate cancer (PC) represents nearly 15% of all new cancer diagnoses (1) and is the most frequently diagnosed cancer in people assigned as male at birth (2). Numerous life-prolonging agents have been approved for the treatment of metastatic castration-resistant PC (mCRPC), notably novel precision medicine approaches including radioligand therapy (RLT) (3). A review of established and novel therapies is beyond the scope of this manuscript, but the current clinical data, including overall survival (OS) rate, is comprehensively reviewed in the 2025 article by Poon et al. (3). Despite these advances in treatment approaches, PC remains one of the leading causes of cancer-related mortality (4). Patients diagnosed with mCRPC have a median OS rate of less than 2 years (5, 6). Furthermore, among those who initiate first-line treatment, there is an approximate 50% reduction in the rate of progression to each subsequent line of therapy due to mortality (7), highlighting the urgent need for improved patient outcomes. As such, understanding factors for disease progression is crucial in this regard; identification and validation of new markers of progression may help to inform treatment selection.

In addition to identifying the likelihood of disease progression, biomarkers may also help predict treatment response and identify patients who are suitable for treatment with specific therapeutic targets. Precision medicine uses prostate-specific membrane antigen (PSMA)-directed radioligand imaging (RLI) to identify patients for treatment with RLT, which binds radioactive molecules directly to the targeted cancer cells (8, 9). PSMA is a transmembrane protein highly expressed at the cell surface of the majority of PCs (10–13) and its expression level is dependent on the disease state, with progressively greater expression reported in mCRPC (14). PSMA has numerous functions within the normal prostate and in PC and is an accessible cell surface target for the delivery of antitumor RLT and other drug therapies (13). Together, these credentials support PSMA as a therapeutic target in mCRPC (13–16).

Diagnostic RLI, such as with the positron emission tomography (PET) imaging agent [^68^Ga]Ga-PSMA-11, can visualize and semi-quantify PSMA expression in tumors, which can then be targeted by treatment with a cytotoxic RLT (8, 9). [^177^Lu]Lu-PSMA-617 is currently the only PSMA-targeted RLT agent approved for use by the Food and Drug Administration (FDA) and the European Medicines Agency in patients with PSMA PET-positive mCRPC who have been treated with androgen receptor pathway inhibitor (ARPI) therapy and for whom it is considered appropriate to delay taxane-based chemotherapy, or patients who have received prior taxane-based chemotherapy (17, 18). [^177^Lu]Lu-PSMA-617 has been shown to improve survival outcomes in patients with mCRPC compared with historical agents; in the pivotal phase 3 VISION trial [^177^Lu]Lu-PSMA-617 plus best standard of care (BSoC), which included historically used approved hormonal treatments, bisphosphonates, radiation therapy, denosumab, or glucocorticoid, improved OS by 4 months compared with BSoC alone (median 15.3 vs 11.3 months; HR, 0.62; 95% CI, 0.52–0.74; *p* < 0.001) (19).

Several other PSMA-targeted RLT strategies are currently under investigation. These include beta-emitters such as [^177^Lu]Lu- PNT2002 (a [^177^Lu]Lu-based PSMA-targeted peptide RLT) (20), I-131-1095 ([^131^I]I-based small-molecule PSMA-targeted RLT) (11) and J591 ([^177^Lu]Lu-J591) (a [^177^Lu]Lu-PSMA-targeted monoclonal antibody) (21). Other RLTs in development include copper-based RLTs, such as [^67^Cu]Cu-SAR-bisPSMA (a [^67^Cu]Cu-based PSMA-targeted RLT with a double PSMA-binding moiety) (22) and alpha-emitters, including [^225^Ac]Ac-J591 (a[^225^Ac]Ac-based PSMA-targeted antibody) (23), [^225^Ac]Ac-PSMA-R2 (a[^225^Ac]Ac-linked PSMA-targeted RLT) (24), [^225^Ac]Ac-PSMA-617 (a[^225^Ac]Ac-linked PSMA-targeted RLT) (25) and [^225^Ac]Ac-PSMA-imaging & therapy (I&T; [^225^Ac]Ac-linked PSMA-targeted RLT used for both imaging and treatment) (26). Discussion on the management of PC with RLT is out of the scope of this manuscript; however, the recently published review article by Almeida et al. provides a comprehensive overview (27).

Although RLI-directed RLT is a promising approach, the expression of PSMA in PC varies both within and between individuals (28, 29). Between 15% and 20% of patients have detectable PSMA-negative metastases on PET scans (19, 30, 31). Even within PSMA-positive mCRPC, the detection and quantification of PSMA heterogeneity may differ based on imaging or immunohistopathology modalities and analysis methods (28). It is likely that the optimal use of a PSMA-targeted RLT as a component of a multi-agent treatment strategy will be needed to account for this therapy-relevant heterogeneity.

**Figure 1:**
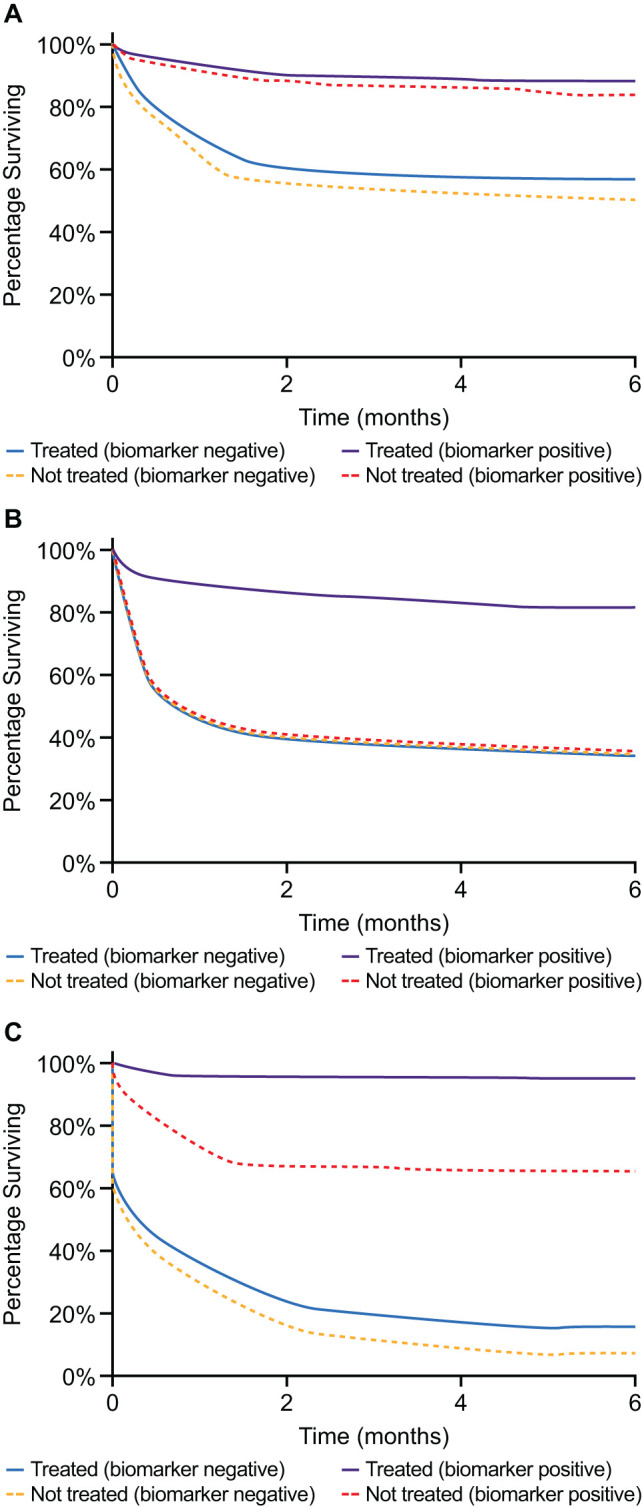
**(A)** Example of a purely prognostic biomarker. Patients with this biomarker have improved survival compared with biomarker-negative patients, independent of treatment. As the treatment effect is the same for both biomarker-negative and biomarker-positive patients, we can surmise that the biomarker is not predictive. **(B)** Example of a purely predictive marker. Biomarker-positive patients display a treatment effect, whereas no treatment effect is observed for biomarker-negative patients. Therefore, treatment effect differs in quality between the groups, suggesting this biomarker is predictive. Note that the untreated biomarker-positive patients have the same survival as the untreated biomarker-negative patients. **(C)** An example of a prognostic and predictive biomarker. The treatment effect is different for biomarker-negative and biomarker-positive patients, suggesting it is predictive. However, biomarker-positive patients have improved survival compared with biomarker-negative patients, independent of treatment group, suggesting it is prognostic.

Given the expanding complexity of the mCRPC treatment landscape, practical guidance on utilizing prognostic and predictive biomarkers to optimize treatment outcomes is urgently required. Prognostic biomarkers can be used to identify the likelihood of a clinical event, such as disease recurrence or disease progression in a particular patient population, and can be identified from trends in observational data (32). Importantly, they can indicate a likely disease outcome independent of the treatment received (33). Conversely, predictive biomarkers are those that are specifically associated with a response to a particular therapy (32, 33). If the treatment effect (experimental vs control) is different for biomarker-positive patients compared with biomarker-negative patients, a biomarker can be considered as predictive (33). To establish whether a biomarker is prognostic or predictive (or both), a formal statistical test between the biomarker and treatment group needs to be performed (33). These concepts are summarized in **Figure 1**. The REMARK guidelines exist to assist in complete and transparent reporting of prognostic studies, such as reporting statistical significance (34).

Several potential prognostic and predictive biomarkers for responses to [^177^Lu]Lu-PSMA-617 treatment in mCRPC have been reported (35–37); however, most of these biomarkers still require standardized and validated measurement methods. This review aims to evaluate the current evidence for, and current clinical applicability of, potential prognostic and predictive biomarkers for [^177^Lu]Lu-PSMA-617 treatment of patients with mCRPC, including imaging, clinical, and molecular biomarkers at varying stages of evaluation, including total tumor and whole-body tumor PSMA expression. The summarized observations of this review are found in **Table 1**.

**Table 1:** Summary of current prognostic and predictive biomarkers for patients with mCRPC, in the context of treatment with [^177^Lu]Lu-PSMA-617.

Biomarker	Role	Summary
**Whole-body PSMA PET** SUV_mean_	Prognostic and predictive	• As a prognostic biomarker: Associated with reduced OS if the whole-body SUV_mean_ is low (< 10), and improved OS if whole-body SUV_mean_ is high (≥ 10), regardless of treatment type (42) • As a predictive biomarker: Whole-body tumor SUV_mean_ values of ≥ 10 were also significantly associated with a favorable PSA response to [^177^Lu]Lu-PSMA-617 treatment compared with cabazitaxel (40) • As a predictive biomarker: Increasing whole-body tumor SUV_mean_ is significantly associated with improved rPFS and OS outcomes with [^177^Lu]Lu-PSMA-617 treatment (85) • Better accounts for lesion heterogeneity than the SUV_max_ (40) • Quantitative PET parameters, including the SUV_mean_, require specialized software not routinely available in most clinics (40) • Currently only assessed with [^68^Ga]Ga-PSMA-11; no large studies have investigated the use of other radioligand tracers for predictive evaluation of the SUV_mean_ in [^177^Lu]Lu-PSMA-617 treatment
**[^18^F]FDG PET** MTV	Prognostic	• Associated with low PSA response rates if high (MTV ≥ 200 mL), regardless of treatment type (40) • [^18^F]FDG PET is widely used in Europe and Australia, but not the US
**SPECT/CT** TTV & NL	Prognostic	• Increases in SPECT TTV and NLs are significantly associated with shorter PSA-PFS, OS (51–53) and PFS (54) • Larger studies to determine appropriate volume cutoffs defining significant increases in SPECT TTV are required (50) • Harmonization of image acquisition and reconstruction between imaging centers are required for results to be reproducible (50)
**Liver metastases**	Prognostic	• Associated with reduced OS (61, 63), BCR (62), and PFS (64)
**CTCs**	Prognostic	• High fraction of PSMA-negative CTCs are associated with reduced PFS and OS (65) • Further validation in a larger cohort of patients is required prior to implementation in clinical practice • Tools to detect PSMA expression in CTCs are not currently used in routine clinical practice, limiting their applicability in the clinical setting
**ctDNA fraction**	Prognostic	• Associated with poor median rPFS, RECIST response rate, and PSA50 outcomes if elevated (68)
**Changes in cancer-associated genes**	Prognostic	• Alterations in *CCNE1, FGFR1, PTEN, RB1, TP53, CDK12, MYC, AR* (and *AR-7* splice variant), and *PI3K* pathway genes are all associated with various negative clinical outcomes (37, 69, 73, 74, 78, 80, 81), including PSA-PFS, PSA50 response, and OS • Larger prospective studies are therefore required to confirm whether these genetic alterations should influence treatment-sequencing decisions in patients with mCRPC
**NLR**	Prognostic	• Associated with reduced OS outcomes if elevated ≥ 2.7 (35) or ≥ 3.75 (82) • Can be readily evaluated from routine blood counts and adopted relatively quickly (83) • Requires further evaluation in trials comparing [^177^Lu]Lu-PSMA-617-treated and untreated/control patients prior to use in clinic
**Hematologic parameters** Neutrophil count Lymphocyte count Pretherapeutic leukocyte count Pretherapeutic lymphocyte count Pretherapeutic platelet count	Prognostic and possibly predictive	• Neutrophil and lymphocyte counts are potential prognostic biomarkers of clinical outcomes (including OS, rPFS, and PSA50), but evaluation of these biomarkers in sufficiently powered clinical studies comparing [^177^Lu]Lu-PSMA-617 and a control arm with BSoC is required to establish their true utility (84) • Pretherapeutic leukocyte, lymphocyte, and platelet counts were all strong predictors (vs osseous tumor volume) of hematotoxicity, both early and late in the treatment course of [^177^Lu]Lu-PSMA-617 (36)
**Whole-body PSMA PET** SUV**_max_**	Possibly predictive	• Nomograms developed from multivariate modeling of data from VISION have identified SUV_max_ as a potentially predictive biomarker (84) • More data are required for validation
**Other potential predictive biomarkers** Time since cancer diagnosis Use of opioid analgesic Presence of PSMA-positive lymph node lesions Hemoglobin levels Aspartate aminotransferase levels Alkaline phosphatase levels PSA50	Possibly predictive Lactate dehydrogenase levels	• All require evaluation and validation in larger biomarker-driven prospective trials comparing [^177^Lu]Lu-PSMA-617 and BSoC before utility as individual predictive biomarkers can be realized (63, 84) • 87.5% of responders to treatment (n = 7/8) achieved PSA50 before their third dose, meaning that monitoring PSA50 levels may assist HCPs in making faster [^177^Lu]Lu-PSMA-617 therapy adjustments (90)

BCR, biochemical response; BSoC, best standard of care; CT, computed tomography; CTC, circulating tumor cell; ctDNA, circulating tumor DNA; FDG, 2-[^18^F]fluoro-2-deoxy-D-glucose; HCP, healthcare professional; MTV, metabolic tumor volume; NLs, new lesions; NLR, neutrophil-to-lymphocyte ratio; OS, overall survival; PET, positron emission tomography; PFS, progression-free survival; PSA, prostate-specific antigen; PSA50, PSA decline ≥ 50%; PSMA, prostate-specific membrane antigen; RECIST, Response Evaluation Criteria in Solid Tumors; rPFS, radiographic PFS; SPECT, single-photon emission CT; SUV_max_, maximum standardized uptake volume; SUV_mean_, mean standardized uptake volume; TTV, total tumor volume.

## 2 Prognostic biomarkers in mCRPC

A number of potential imaging and clinical prognostic biomarkers for patients with PSMA-positive mCRPC have been explored.

### 2.1 Total tumor PSMA expression

When evaluating the expression of PSMA throughout the body using PET imaging, a radioactive ligand (such as [^68^Ga]Ga-PSMA-11) that targets PSMA is used (38). The radioactive tracer emits positrons that produce annihilation photons, which are in turn detected by the PET scanner, allowing a three-dimensional image of the tracer’s loci inside the body to be rendered (39). As an indicator of PSMA expression, the mean standardized uptake value (SUV_mean_) is a measurement parameter used in PET scans that quantifies the average concentration of radiotracer ligand uptake within the entire segmented tumor volume, whereas the maximum standardized uptake value (SUV_max_) is the SUV of the single voxel with the highest concentration of radiotracer ligand in the entire segmented tumor volume (40).

Current evidence suggests that PSMA levels may serve as a prognostic biomarker for patients with mCRPC, and one team of investigators recently released research showing that prognostic PSMA PET nomograms based on the PROMISE study criteria had prognostic utility across early and late stages of PC (41). The open-label, randomized, phase 2 TheraP study (NCT03392428) was the first ever randomized trial comparing [^177^Lu]Lu-PSMA-617 to the BSoC treatment for patients with mCRPC, with 200 eligible participants with PSMA-positive mCRPC randomly assigned (1:1) to treatment with either [^177^Lu]Lu-PSMA-617 (n = 99) or cabazitaxel (n = 101) (30, 40). In an updated OS analysis, no significant differences in mean OS were observed between the [^177^Lu]Lu-PSMA-617 and cabazitaxel arms (19.1 vs 19.6 months, respectively), irrespective of baseline SUV_mean_ (40). Furthermore, patients with a high whole-body SUV_mean_ (≥ 10) had improved OS outcomes, regardless of treatment type, compared with patients with an SUV_mean_ < 10 (42), though patients with an SUV_mean_ < 10 treated with [^177^Lu]Lu-PSMA-617 were still likely to benefit from longer radiographic progression-free survival (rPFS) compared with those treated with cabazitaxel (40). These data are important, as they come from a study for which we have the longest RLT versus BSoC follow-up data. The exploratory secondary analysis of the phase 3 VISION study (NCT03511664), in which eligible participants were randomized 2:1 to receive [^177^Lu]Lu-PSMA-617 therapy plus BSoC (n = 548) or BSoC alone (n = 278), also provides evidence of the prognostic utility of SUV_mean_ in patients with mCRPC (43). Here, median OS was longest in patients from the BSoC alone arm who were in the highest whole-body tumor SUV_mean_ quartile, suggesting that higher mean PSMA expression could be a favorable prognostic factor for survival in patients with mCRPC (43). However, it is worth noting that while median OS was longest in the highest SUV_mean_ quartile of the BSoC arm, the median OS in the lower three SUV_mean_ quartiles were very similar (43). Furthermore, high SUV_mean_ was not associated with improved median rPFS in the BSoC only arm; rPFS in this arm remained approximately the same regardless of SUV_mean_ quartile (43), casting doubt on the value of SUV_mean_ as a prognostic biomarker in the BSoC arm.

These results suggest that the SUV_mean_ on treatment with [^68^Ga]Ga-PSMA-11 may be a prognostic factor for OS, independent of the treatment received (42). Investigators have commented that SUV_mean_ gives an evaluation of tumor status that better accounts for therapeutically relevant intra- and interlesional PSMA heterogeneity, compared with SUV_max_ (40). However, it is important to note that SUV_mean_ is not routinely reported in PSMA PET scan reports currently, and the implications this may have on its adoption in clinical practice are covered in the discussion section below.

### 2.2 2-[^18^F]fluoro-2-deoxy-D-glucose PET metabolic tumor volume

Imaging with 2-[^18^F]fluoro-2-deoxy-D-glucose ([^18^F]FDG) PET is a well-established and widely studied imaging tool assessing glucose metabolism in a variety of malignancies (44, 45). Investigators in a single-center, prospective, phase 2 trial observed that patients with mCRPC who have high intensity radioligand uptake at disease sites on [^18^F]FDG PET imaging may have poorer OS (46). The prognostic value of metabolic tumor volume (MTV), which refers to the metabolically active volume of the tumor segmented by [^18^F]FDG PET (47), was therefore examined in a *post hoc* biomarker analysis of the phase 2 TheraP study (40). The investigators who conducted this *post hoc* analysis observed that a high volume of [^18^F]FDG PET MTV (i.e. an MTV ≥ 200 mL) was associated with a lower prostate-specific antigen (PSA) response rate (vs MTV < 200 mL) for both treatment groups (RLT and cabazitaxel) combined (n = 200). Of note, patients were not included in the TheraP study if they presented with site(s) of discordant disease (i.e. sites of disease that are [^18^F]FDG-positive with minimal PSMA expression, defined as [^18^F]FDG intensity > [^68^Ga]Ga-PSMA activity or [^68^Ga]Ga-PSMA SUV_max_ < 10), so these findings must be viewed through the lens of patients without sites of discordant disease (40). Similar results were also observed by the investigators conducting the phase 2 LuPSMA trial, who found that [^18^F]FDG-positive MTV, when measured by [^18^F]FDG PET, had prognostic significance for the survival of patients with mCRPC (46). However, additional data from a larger patient group are required to fully evaluate the applicability of this method for prognostic evaluation in mCRPC in a routine clinical setting. It should also be noted that while [^18^F]FDG PET imaging is widely used in Europe and Australia for mCRPC, in the US, PSMA PET-only imaging criteria are predominantly used for identifying patients suitable for [^177^Lu]Lu-PSMA-617, based upon the eligibility criteria of the VISION study (48). This discrepancy should be taken into account by physicians in countries where FDG PET is not routinely used.

### 2.3 [^177^Lu]Lu-PSMA-617 single-photon emission computed tomography/computed tomography

Single-photon emission computed tomography (SPECT)/computed tomography (CT) allows lesions visualized by functional imaging to be correlated with anatomic structures, resulting in increased sensitivity and specificity of scintigraphic findings (49). The uptake of [^177^Lu]Lu-PSMA-617 by tumor cells can be determined using SPECT and quantified as SPECT total tumor volume (TTV) (50). Increased SPECT TTV and new lesions (NLs) in early treatment cycles (1, 2) have been associated with reduced OS and PSA progression-free survival (PSA-PFS) in retrospective studies (51–53), showing promise as early prognostic biomarkers during PSMA-targeted RLT. In another retrospective analysis of 127 patients evaluated for change in SPECT/CT TTV at the week 6 (dose 2) timepoint, an increase in SPECT/CT TTV was found to potentially predict reduced PFS (54). Increased TTVs and NLs at the start of cycles 2 and 3, as determined by SPECT/CT, have also been shown to be independently associated with increased risk of death in a recently published retrospective study (52).

SPECT/CT performed 24 hours after treatment with [^177^Lu]Lu-PSMA-617 has been shown to lead to changes in treatment management in 49% of patients (n = 60/122), highlighting its therapeutic value (52, 55). Importantly, tailoring RLTs based on early biomarker response (such as PSA response and changes in TTV and NLs) has also been shown to maintain comparable outcomes to continuous treatment, while allowing for the benefit of a treatment vacation (53). Dosimetric parameters on SPECT/CT have also been investigated in a retrospective study, which found baseline lesion-absorbed dose of [^177^Lu]Lu-PSMA-617 was significantly correlated with PSA response, though SPECT/CT lesion-based responses had a limited relationship with absorbed dose (56). New generation multi-detector cadmium-zinc-telluride (CZT)-based SPECT/CT that can acquire scans much faster than current modalities has been shown to have comparable detection/targeting rates compared to the commonly used SPECT/CT systems (Discovery 670 Pro) (57). Furthermore, SPECT/CT at 4 hours post–[^177^Lu]Lu-PSMA-617 treatment shows promise as an alternative to 24-hour post-treatment SPECT/CT for assessing treatment response (58).

These results emphasize the practicality of using SPECT/CT for treatment response assessment, highlighting the potential utility of SPECT/CT as a prognostic tool for establishing likely disease outcomes in patients with mCRPC. However, these findings need to be validated in larger cohorts of patients and further research is needed to ensure the practical applicability of SPECT/CT, including appropriate volume cutoffs to define significant increases in SPECT TTV and harmonization of image acquisition and reconstruction between imaging centers and systems (50).

### 2.4 Liver metastases

The liver has a critical role in the metabolism, detoxification, and synthesis of a number of proteins and factors vital for fluid balance and blood clotting, and it is hypothesized that liver metastases contribute to cancer mortality through these vital physiological functions (59). In a meta-analysis of 83 studies evaluating 604,853 patients treated for a wide variety of cancer types, liver metastases were shown to negatively impact OS (HR, 1.77; 95% CI, 1.62–1.93) (59). Liver metastases have also been associated with poor OS outcomes in mCRPC, regardless of treatment type (60). Of therapeutic relevance, liver metastases can also have heterogeneous levels of PSMA in mCRPC (31).

The prognostic utility of the presence of liver metastases, visualized by RLI, has been investigated in a number of observational and retrospective studies in patients with mCRPC treated with [^177^Lu]Lu-PSMA-617 (61–64). In the WARMTH multicenter retrospective analysis, the presence of PSMA-positive bone and liver metastases, as well as poor Eastern Cooperative Oncology Group performance status, were shown to be significantly associated with worse OS in univariate and multivariate analyses (61). A multicenter retrospective study of 145 patients with mCRPC also indicated a positive relationship between the presence of PSMA-positive visceral metastases and reduced biochemical response, as defined by a PSA decline of ≥ 50% (PSA50) from baseline to at least 2 weeks after the start of treatment with [^177^Lu]Lu-PSMA-617 (p < 0.01) (62). In a single-center retrospective analysis of 52 patients with mCRPC treated with [^177^Lu]Lu-PSMA-617, the presence (vs absence) of PSMA-positive liver metastases was also associated with poorer OS (HR, 6.981; 95% CI, 2.583–18.863; p < 0.001) (63). Furthermore, in a prospective observational study (n = 52), presence of PSMA-positive liver metastases was also associated with reduced PFS in patients treated with [^177^Lu]Lu-PSMA-617 (64).

These studies highlight that the presence of PSMA-positive liver metastases may have a role as a prognostic biomarker that could be applied in clinical practice, as they indicate individuals with likely poor clinical outcomes who may benefit from [^177^Lu]Lu-PSMA-617 treatment. However, further evaluation is needed to prospectively determine their application in the clinical setting among patients with mCRPC.

### 2.5 Circulating tumor cells

While targeted imaging provides an opportunity for whole-body phenotyping of PSMA expression at the macroscopic level, liquid biopsy testing, specifically of circulating tumor cells (CTCs), could offer an alternative method of PC disease identification and characterization (65). The role of PSMA-positive (vs PSMA-negative) CTCs as a potential prognostic biomarker in the context of RLT was suggested by the results of a prospective proof-of-concept study in patients (n = 20) with advanced mCRPC (65). Here, the investigators found that a high fraction of PSMA-negative CTCs (high fraction: mean 54%, vs low fraction: mean 19%) was prognostic for both shorter PFS (odds ratio [OR], 1.236; 95% CI, 1.035–2.587; p = 0.0043) and shorter OS (OR, 1.056; 95% CI, 1.008–1.141; p = 0.0182) in patients treated with [^177^Lu]Lu-PSMA-617 (65). However, as commented by the investigators, this was a small, single-center, proof-of-principle study with only moderate statistical power, and further validation in a larger cohort of patients is required prior to implementation in clinical practice (65). Furthermore, tools to detect PSMA expression in CTCs are not currently used in routine clinical practice, limiting their applicability in the clinical setting.

### 2.6 Circulating tumor DNA fraction

In patients with cancer, tumor DNA is often shed into the blood and mixed with cell-free DNA from leukocytes that have undergone apoptosis (66). The prognostic utility of circulating tumor (ct)DNA and ctDNA fraction in plasma has been seen across several cancer types including mCRPC (67). ctDNA fraction can be associated with tumor burden and can help assess the likely disease trajectory of patients across different cancer types and clinical scenarios (66). In a real-world outcomes study conducted in the US, the prognostic value of ctDNA fraction was assessed in patients undergoing liquid biopsy testing for four cancer types including mCRPC (67). In this study, the investigators observed that a ctDNA fraction ≥ 10% was highly correlated with OS in a univariable analysis of patients with mCRPC (HR, 3.3; 95% CI, 2.04–5.34; p < 0.001), regardless of their treatment type (67). Furthermore, this finding was consistently seen in patients with all of the other cancer types evaluated, leading the investigators to conclude that plasma ctDNA tumor fraction is an independent prognostic biomarker, with the potential to guide clinical conversations around expected disease trajectory and, therefore, treatment selection (67). However, the investigators did also note that its predictive utility is still to be proven. Thus, the use of ctDNA tumor fraction or changes in ctDNA fraction that occur on therapy as a guide to optimize treatment outcomes in patients with mCRPC requires validation in prospective clinical trials before it can be incorporated into clinical practice (67).

In the context of [^177^Lu]Lu-PSMA-617 treatment, associations between baseline ctDNA levels and outcomes have been assessed as an exploratory analysis of the phase 3, open-label, randomized PSMAfore study (NCT04689828) (68). Of the 360 samples collected from 468 patients, investigators evaluated ctDNA levels, and observed increased ctDNA levels were associated with worse median rPFS, Response Evaluation Criteria in Solid Tumors response rate, and PSA50 outcomes, regardless of therapy (68). It was noteworthy that patients with this negative prognostic biomarker still had longer median rPFS outcomes with [^177^Lu]Lu-PSMA-617 treatment compared with ARPIs (68). In a single-center observational cohort study (n = 46), higher levels of ctDNA were significantly correlated with shorter PFS in patients treated with [^177^Lu]Lu-PSMA-617 (69). The investigators found that a quartile index stratification of ctDNA levels identified three prognostic groups (low/undetectable, intermediate, and high ctDNA) with different Kaplan–Meier PFS estimates (median 7.3 vs 4.3 vs 2.4 months*;* p = 0.0023), though they also noted the small size of their study as a limitation (69). Similar findings were observed in a recently presented exploratory analysis of patients enrolled in the phase 2 TheraP trial (70). Here, the investigators analyzed baseline blood samples from 178 participants who received ≥ 1 cycle of protocol-assigned treatment, finding that the odds of a PSA50 response to [^177^Lu]Lu-PSMA-617 in patients with ctDNA < 2% were significantly higher than in those patients treated with cabazitaxel (p = 0.0067) (70). Furthermore, higher ctDNA fractions were associated with shorter PFS in patients treated with [^177^Lu]Lu-PSMA-617 (p < 0.001), though not cabazitaxel (p = 0.35) (70). The investigators concluded that ctDNA fraction is a candidate prognostic and predictive biomarker for differential response to [^177^Lu]Lu-PSMA-617 treatment versus taxane chemotherapy in molecular imaging-identified patients with mCRPC who have progressed after docetaxel (70).

### 2.7 Changes in cancer-associated genes

In addition to quantifying tumor fraction, ctDNA analysis can detect specific genomic aberrations, including those involving DNA-repair genes (such as *BRCA1*, *BRCA2*, *ATM*, *PALB2*, *FANCA*, *RAD51D*, *CHEK2*, and *CDK12*) (71). In a 2023 study, pretreatment mutations, deletions, fusions, and amplifications of 83 cancer-associated genes were evaluated in the ctDNA of 44 patients with PSMA-positive mCRPC prior to therapy with PSMA-targeted RLT ([^177^Lu]Lu-PSMA-I&T and [^177^Lu]Lu-PSMA-617) (37). Non-responders (defined as PSA response < 50%) were significantly more likely to have amplifications of the cancer-related genes *CCNE1* and *FGFR1* prior to treatment (37). However, the investigators did note that additional studies to confirm these findings are required (37). Interestingly, alterations in DNA-repair genes were associated with an improved PSA50 response rate following treatment with [^177^Lu]Lu-PSMA-617, suggesting that the higher PSA50 response rates observed in the DNA-repair cohort might be due to an increased sensitivity to ionizing radiation (72).

Alterations in the tumor suppressor genes *PTEN*, *RB1*, and *TP53* were found to be significantly associated with poorer outcomes (including PSA-PFS and OS) in a study of ctDNA in 32 patients treated with [^177^Lu]Lu-PSMA-617 plus intermittent treatment with the small molecule transduction inhibitor idronoxil (73). As these data came from a small, single-center study, the investigators commented that further studies are required to fully establish the potential of these genetic variants as prognostic biomarkers for [^177^Lu]Lu-PSMA-617 RLT (73). Similarly, in the PSMAfore study, median rPFS was also observed to be significantly shorter for patients with detected versus undetected alterations in key PC genes, such as the androgen receptor gene (*AR*) and *TP53*, suggesting a potential for prognostic significance (68).

In a recently published retrospective study characterizing molecular prognosticators of benefit to [^177^Lu]Lu-PSMA-617 among patients with mCRPC (n = 115), the investigators observed that baseline alterations in *CDK12*, *MYC*, and *FGFR* were associated with a reduced PSA50 response after [^177^Lu]Lu-PSMA-617 treatment (74). Similarly, another group study assessing genomic alterations in 120 patients with mCRPC found that amplification of genes regulating cell-cycle pathways were associated with poorer OS (75). Conversely, another study in 71 patients with mCRPC found there were no significant associations between [^177^Lu]Lu-PSMA-617 outcomes and genomic alterations (in genes including *ATM, ATR, BRCA1/2, BRIP1, CHEK2, EPCAM, FANCA, GEN1, HOXB13, MLH1, MSH2/6, NBN, PALB2, PMS2, RAD51C, RAD51D*, and *TP53*), or prespecified pretreatment PET parameters (76). However, in the Phase 2 TheraP trial, ATM loss in baseline ctDNA was associated with more favorable outcomes in select patients treated with [¹⁷⁷Lu]Lu–PSMA-617 (70). Properly powered prospective studies specifically designed to determine links between somatic gene alterations and treatment-sequencing efficacy are required to apply the findings clinically. Given that these studies were conducted in patients treated with [^177^Lu]Lu-PSMA-617, further validation is needed on whether these genomic alterations are predictive or prognostic.

Amplifications, mutations, and splice variants involving the *AR* gene are commonly seen in mCRPC (77). In a genomic analysis of 101 CRPC metastases, a region approximately 624 kb upstream of the *AR* gene was a commonly observed site of structural DNA variation (78). *AR* amplification occurred in 70% of analyzed cases and was associated with significantly elevated *AR* mRNA expression (p = 9 × 10^−8^) (78). This is consistent with previous research showing that genomic aberrations in *AR* are rare in localized PC but reasonably common in CRPC (6) and are an acquired mechanism of resistance to ADT and ARPIs (79). Other study groups have since conducted studies on how *AR* gene alterations and changes in gene expression could act as potential prognostic biomarkers in mCRPC (69, 80, 81). In one single-center, open-label, phase 2 trial (NCT03454750) evaluating the activity of [^177^Lu]Lu-PSMA-617 and pretreatment plasma *AR* DNA copy number in patients with mCRPC, patients who gained copies of the *AR* gene in their plasma were more likely to develop early progressive disease compared with patients with no gain of *AR* gene copies (p = 0.0002) (80). Patients with these *AR* gene amplifications had a shorter median OS compared with those who did not have these amplifications (7.4 vs 19.1 months respectively; p = 0.020) (80). The researchers concluded that *AR* status could therefore indicate cases of mCRPC with early resistance to [^177^Lu]Lu-PSMA-617 (80). In a single-center observational cohort study of 57 patients with late-stage mCRPC treated with a different PSMA-targeted RLT, [^177^Lu]Lu-PSMA-I&T (69), structural rearrangements in the *AR* gene and gene alterations in the *PI3K* pathway were observed to be independently associated with poor prognosis, and patients with these changes did not gain lasting benefit from [^177^Lu]Lu-PSMA-I&T (69). However, the investigators stated that prospective evaluation in larger biomarker-driven trials is warranted (69). In the PSMAfore study, median rPFS was significantly reduced in patients with detected versus undetected *AR* gene alterations (68). Moreover, a prospective analysis of patients (n = 19) undergoing [^177^Lu]Lu-PSMA-617 therapy for mCRPC suggested that for both full-length *AR* and the truncated *AR* splice variant, *AR*-V7, mRNA copy numbers might serve as prognostic biomarkers for high tumor burden in patients with mCRPC prior to initiating [^177^Lu]Lu-PSMA-617 treatment, although further study is required (81). Overall, these studies indicate that alterations in *AR, ATM, CCNE1, FGFR1, PTEN, RB1, TP53, CDK12, MYC, and FGFR* genes may serve as prognostic biomarkers for mCRPC, including in those treated with [^177^Lu]Lu-PSMA-617, though as noted in many of these studies, further research to validate these findings is required.

### 2.8 Neutrophil-to-lymphocyte ratio

The neutrophil-to-lymphocyte ratio (NLR) is thought to be a marker of inflammation, host immune response, and the tumor microenvironment in patients with a wide variety of cancers (82, 83). The value of the NLR in prognosticating [^177^Lu]Lu-PSMA-617 treatment outcomes in mCRPC has thus been explored in two retrospective studies (35, 82). In the first, a retrospective analysis of data from 180 patients with mCRPC treated in sequential prospective RLT trials (utilizing [^177^Lu]Lu‐J591, [^90^Y]Y‐J591, [^177^Lu]Lu‐PSMA‐617, or [^225^Ac]Ac‐J591), a median NLR of ≥ 3.75 (vs < 3.75) was found to be significantly associated with a worse OS (HR, 1.06; 95% CI, 1.02–1.09; p = 0.002), regardless of treatment type (82). In the second retrospective study in 61 patients with mCRPC treated with [^177^Lu]Lu-PSMA-617, high NLR (≥ 2.7) demonstrated the strongest (of five hematologic and inflammatory parameters) association with shorter OS (HR, 3.32; 95% CI, 1.66–6.65; p = 0.001) (35). However, as noted by the investigators, the prognostic utility of NLR requires further evaluation in trials comparing [^177^Lu]Lu-PSMA-617-treated and control patients. Should additional research support its use as a prognostic (or predictive) biomarker, NLR may be a highly useful biomarker for the clinical setting, as it can be readily evaluated from routine blood counts and so may be widely adopted relatively quickly (83).

### 2.9 Hematologic parameters

Nomograms developed from multivariate modeling of data from the phase 3 VISION study identified neutrophil count as a potential prognostic biomarker for OS and lymphocyte count as a potential prognostic biomarker of OS, rPFS, and PSA50 in patients treated with [^177^Lu]Lu-PSMA-617 (84). Further evaluation of these biomarkers in a sufficiently powered clinical study comparing [^177^Lu]Lu-PSMA-617 and a control arm with BSoC is required to firmly establish their prognostic (and possibly predictive) utility.

## 3 Predictive biomarkers for response to [^177^Lu]Lu-PSMA-617 therapy in PSMA-positive mCRPC

The use of imaging and clinically based biomarkers is being evaluated for its predictive value in determining response to [^177^Lu]Lu-PSMA-617 therapy in patients with PSMA-positive mCRPC. Understanding the role these biomarkers have in predicting patient response to treatment is a vital tool for optimizing treatment selection in an increasingly complex landscape. Below, we discuss the latest evidence for the utility of various biomarkers for predicting response to [^177^Lu]Lu-PSMA-617 treatment in patients with mCRPC. It should also be noted that some biomarkers, such as whole-body PSMA levels, may have both prognostic and predictive utility.

### 3.1 Whole-body tumor PSMA expression

PSMA levels, as determined by the SUV_mean_ of PSMA PET, were evaluated in *post hoc* analyses of two pivotal clinical trials of [^177^Lu]Lu-PSMA-617 in mCRPC (40, 85). In the phase 2 TheraP study (n = 200) the investigators utilized prespecified cutoff points for SUV_mean_, observing that whole-body tumor SUV_mean_ values of ≥ 10 were significantly associated with a favorable PSA response to [^177^Lu]Lu-PSMA-617 treatment compared with cabazitaxel (OR, 12.19; 95% CI, 3.42–58.76 *vs* 2.22; 95% CI, 1.11–4.51; p*_adj_* = 0.039 for treatment-by-SUV_mean_ interaction) (40). Notably, however, 57 patients harboring tumors with SUV_mean_ values < 10 also showed radiographic responses to [^177^Lu]Lu-PSMA-617 treatment (40). In an exploratory *post hoc* subgroup analysis (n = 548) of the VISION study, increasing whole-body tumor SUV_mean_ was significantly associated with improved rPFS and OS outcomes with [^177^Lu]LuPSMA–617 treatment (43, 85). In the [^177^Lu]Lu-PSMA-617 plus BSoC treatment arm, every one-unit increase in whole-body tumor SUV_mean_ was associated with a 12% decrease in the risk of an rPFS event and a 10% decrease in the risk of death (43). It is important to note that in this study, no cutoff or threshold SUV_mean_ was identified, as survival was linearly correlated with whole-body SUV_mean_ (85, 86). Nomograms developed from multivariate modeling of data from 831 patients in the phase 3 VISION study, who received [^177^Lu]Lu-PSMA-617 plus BSoC or BSoC alone, also identified SUV_max_ as a potentially predictive biomarker (84). However, evaluation of SUV_max_ in a sufficiently powered randomized clinical study comparing [^177^Lu]Lu-PSMA-617 and BSoC is required to firmly establish its predictive potential.

Importantly, the quantitative PET parameters used in these exploratory [^177^Lu]Lu-PSMA-617 studies require specialized software, and the resources required to routinely assess whole-body tumor SUV_mean_ are not available in most clinics (40); hence, the clinical applicability of SUV_mean_ as a prognostic or predictive biomarker may be limited at this time. However, it should be noted that a four-category score that incorporates both heterogeneity and intensity of tumors, derived from tools on a standard PET workstation, was found to be comparable with quantitative SUV_mean_ in terms of predictive utility for response to [^177^Lu]Lu-PSMA-617 treatment (87). In addition, these studies utilized PSMA PET imaging with [^68^Ga]Ga-PSMA-11, which is currently the only radiopharmaceutical approved by the FDA for assessing PSMA-positive PC lesions (88), although current NCCN Clinical Practice Guidelines in Oncology (NCCN Guidelines^®^) and American Society of Clinical Oncology guidelines state that all FDA-approved radioligand tracers (namely [^68^Ga]Ga-PSMA-11, [^18^F]DCFPyL, and [^18^F]rhPSMA-7.3) can be used for identifying suitable patients (71, 89). To our knowledge, there are no large studies that have investigated the use of other radioligand tracers for predictive evaluation of SUV_mean_ in [^177^Lu]Lu-PSMA-617 therapy.

In addition, the caveats noted above regarding the routine availability of specialized resources required to evaluate SUV_mean_ (40) could also apply to SUV_max_, thus limiting the widespread clinical adoption of SUV evaluations at this time.

### 3.2 Hematologic parameters

The value of hematologic parameters for prediction of [^177^Lu]Lu-PSMA-617 treatment hematotoxicity was also explored in a retrospective study of 67 patients (36). In this study, the investigators compared pretherapeutic hematologic parameters for prediction of hematotoxicity after [^177^Lu]Lu-PSMA-617 treatment. The study found that pretherapeutic leukocyte, lymphocyte, and platelet counts were all strong predictors (vs osseous tumor volume) of hematotoxicity both early and late in the treatment course (36). This study suggests that hematological parameters may have potential as predictive biomarkers, though their utility needs to be formally validated in a prospective randomized controlled trial.

### 3.3 Other potential predictive biomarkers

Development of the nomograms outlined at the beginning of this section identified a number of other primarily clinical biomarkers with predictive potential in [^177^Lu]Lu-PSMA-617 therapy (84), including time since cancer diagnosis; use of opioid analgesic; presence of PSMA-positive lymph node lesions; and levels of hemoglobin, aspartate aminotransferase, lactate dehydrogenase, and alkaline phosphatase (63, 84). Again, these would require evaluation and validation in larger biomarker-driven prospective trials comparing [^177^Lu]Lu-PSMA-617 and BSoC before they could be utilized as individual predictive biomarkers. Furthermore, a recent real-world retrospective cohort study has shown that monitoring PSA50 levels in patients with mCRPC during treatment with [^177^Lu]Lu-PSMA-617 may assist clinicians in making faster therapy adjustments, as 87.5% of responders to treatment (n = 7/8) achieved PSA50 before their third dose (90).

## 4 Forecasting models

In the absence of clinically validated prognostic and predictive biomarkers, nomograms are among the most commonly used tools to estimate prognosis in oncology and medicine. They function by generating an individual numerical probability of a clinical event by integrating diverse prognostic and determinant variables (91). In a recent multicenter, retrospective study, data from 2414 patients were assessed to compare the prognostic value of PSMA PET staging, categorized by PROMISE criteria, with established clinical nomograms (92). Imaging, clinical, and follow-up data were collected, and PSMA PET data were then compared with established clinical risk scores for prediction of OS in patients at all stages of PC (92). The investigators found that their nomograms accurately stratified high-risk and low-risk groups for OS in early and late stages of PC, yielding equal or better prediction accuracy compared with established clinical risk tools (92).

Externally validated nomograms prognostic for mCRPC and predictive of response to [^177^Lu]Lu-PSMA-617 treatment in patients with mCRPC have been developed for clinicians in institutions where PSMA-targeted RLT has been introduced as a novel therapeutic option (84, 93). Gafita et al. have developed three externally validated nomograms that assess OS, PSA-PFS, and PSA50 in males with mCRPC receiving [^177^Lu]Lu-PSMA-617, concluding that these prognostic models may assist in clinical trial design and individual clinical decision-making (93). Nomograms developed from multivariate modeling of data from 831 patients in the phase 3 VISION trial identified a number of parameters with potentially predictive value, such as PSMA expression (e.g. SUV_mean_) and lymphocyte count (84). However, it should be noted that these nomograms utilized [^68^Ga]Ga-PSMA-11 for patient identification (84, 93) and have not been validated with other radiotracers. Furthermore, while the predictive value of these nomograms was alluded to by the investigators for one of these models (93), formal statistical validation is difficult, because a comparator arm of patients that had not received [^177^Lu]Lu-PSMA-617 was not included in the study design. Therefore, before their use in clinical practice can be widely adopted, clinicians must adequately understand the assumptions and limitations of available nomograms, specifically regarding their ability to provide real-time prognostic information and to predict disease recurrence (91). Moreover, many clinical centers may not have the necessary resources to measure some of the parameters included in the available nomograms.

## 5 Discussion

This review examines and summarizes data for a number of potential prognostic and predictive biomarkers for [^177^Lu]Lu-PSMA-617 treatment in patients with PSMA-positive mCRPC. While there are some promising candidates for prognostic and predictive biomarkers in this setting, further research is broadly needed to fully elucidate their utility in aiding treatment decision-making. The development of accurate tools for establishing the likelihood of disease progression and/or treatment response will provide invaluable assistance to clinicians in making optimal treatment-sequencing decisions, for example, where patients have shown an apparent “complete response” to treatment or where PSMA expression has become downregulated after treatment. Disease progression, or the development of adverse events, are also important considerations for continuing or initiating therapy. Moreover, further research is still needed to determine the right course of action when biomarkers are discordant, such as when serum PSA and whole-body PSMA PET SUV_mean_ measurements are trending in opposite directions after therapy.

The boundary between defining a biomarker as predictive versus prognostic is often confusing, highlighting the importance of appropriately designed clinical trials and validation processes to identify the role of biomarkers as prognostic and/or predictive. Uncertainty around the prognostic or predictive value of a biomarker can arise from inadequate reporting of the criteria used to determine the prognostic/predictive value of factors (94, 95). Furthermore, it is important to recognize that selective and incomplete reporting of prognostic factors (such as the use of preferred definitions and omitting data) can lead to inflated conclusions (96). The REMARK guidelines should be utilized in the design of prognostic studies to ensure sufficient study quality and transparent reporting (34). There are several common limitations in study designs which further complicate interpretation of biomarker study data. Firstly, although studies that assess and compare treatment outcomes in biomarker-positive versus biomarker-negative patients are informative, they often neglect to include a comparator arm containing patients receiving a different treatment regimen (e.g. BSoC). This is important for ascertaining predictive utility, as a comparator arm is required for a formal statistical test for interaction between the treatment and biomarker (33).

Furthermore, inconsistent terminology in the literature, such as interchangeability between the use of “prognostic” and “predictive”, can cause confusion (33), especially when some biomarkers may be both prognostic and predictive (such as SUV_mean_). For instance, several articles cited in this review discuss the prognostic utility of a biomarker in predicting treatment response to [^177^Lu]Lu-PSMA-617, and so are therefore describing a predictive biomarker. Furthermore, to determine whether a biomarker is purely prognostic, a significant association between biomarker and outcomes needs to be established (regardless of treatment type), and it must be shown that treatment effects do not depend on the biomarker (33). Future clinical trials that attempt to elucidate the prognostic and/or predictive utility of specific biomarkers must take the above points into consideration if they wish to be optimally informative.

Much work is still required in order to validate accurate prognostic biomarkers for patients with mCRPC. With regard to SUV_mean_ as a prognostic biomarker, although trial data provide evidence of poorer survival outcomes in patients with a lower SUV_mean_, regardless of their treatment (42), the resources such as the specialized software required to assess whole-body SUV_mean_ are not routinely available in current clinical practice (40). This highlights an unmet need to validate existing software in large clinical trials, widen its availability, and provide training that will encourage its use among healthcare professionals (HCPs). Having said this, visual overall assessments of whole-body PSMA PET scans to determine the level of uptake can still be undertaken and may assist HCPs in gauging the spread of disease. Notably, [^68^Ga]Ga-PSMA-11 is currently the only radiopharmaceutical approved by the FDA for imaging PSMA-positive lesions in patients with mCRPC (88), although current guidelines allow for use of other PSMA radiotracers thus far, based on their observed near-equivalence to [^68^Ga]Ga-PSMA-11 (71, 89). In terms of utilizing [^18^F]FDG PET for evaluation of MTV and its influence on disease trajectory, it was observed that high [^18^F]FDG PET MTV was associated with lower treatment response (measured by PSA levels), regardless of treatment type (40). [^18^F]FDG-derived MTV is a promising candidate for a prognostic biomarker in patients with mCRPC, especially in countries (typically in Europe and Australia) that more routinely perform [^18^F]FDG PET in mCRPC.

Among other points to consider regarding PSMA PET imaging is that PSMA is regulated in part by the AR signaling pathway (13, 97, 98). PC cells can upregulate PSMA after acute AR blockade (99), while downregulation of PSMA is associated with tumor response to AR-directed treatment (100), and tumors can further upregulate PSMA at the time of AR-reactivation and treatment resistance (101). Loss of AR expression or AR signaling dependence in a subset of CRPC tumors, including those that develop neuroendocrine features, may account for variations in PSMA expression or loss of PSMA-positivity that can be seen in some cases of CRPC (31, 98). More research is thus needed to fully understand how AR signaling interactions affect PSMA protein levels and how serial PET imaging can dynamically capture this process. Furthermore, other mechanisms beyond AR signaling can also regulate PSMA expression, though these are still yet to be fully understood (98). Regardless, PSMA has been demonstrated to be a functionally relevant progression marker of PC in most patients, highlighting that imaging PSMA with PSMA PET to determine the optimal timing of treatment may be of paramount importance.

SPECT/CT measurement of [^177^Lu]Lu-PSMA-617 uptake has also shown some promise as a biomarker prognostic for disease outcomes (50–53, 55), though the current data are limited and so larger studies and standardization are required for its validation. Though liver metastases are an established prognostic biomarker for poor clinical outcomes in advanced mCRPC (60), there is still a need for further prospective studies to generate data that can determine the extent that patients with liver metastases would benefit from [^177^Lu]Lu-PSMA-617 treatment. However, if a patient presents with advanced mCRPC and liver metastases, their prognosis is poor, and so there may be an inclination to treat these patients with whatever treatment options remain. ctDNA fraction and CTCs also suggest promise as prognostic biomarkers (37), but again, studies with larger patient populations are required to elucidate whether these can be applied in the clinical setting at this stage, and whether dynamic changes in these parameters can be useful for treatment decision-making. Likewise, preliminary findings on specific cancer-related gene mutations and amplifications in ctDNA also point to alterations with potential prognostic utility (37, 66, 67), although again, further studies are required to establish their clinical utility when detected before or during [^177^Lu]Lu-PSMA-617 therapy. Taken together, the above points suggest that while there are several very promising biomarkers with potential prognostic utility, such as CTCs and ctDNA fraction, they all require further validation prior to use in clinic. However, some prognostic biomarkers such as [^18^F]FDG MTV and PSMA PET can potentially be adopted in clinic now, though their uptake may be limited by the factors discussed above. Of note, in addition to clinical decision-making, emerging biomarkers may also help understand the biologic mechanisms underlying primary and acquired resistance to PSMA-targeted RLT that could guide future combination treatment strategies.

While many of the data have been focused on the prognostic utility of these aforementioned biomarkers, there is also an unmet need to understand their predictive utility, as this informs on how particular biomarkers may influence/predict treatment outcomes with [^177^Lu]Lu-PSMA-617, thus aiding optimal treatment selection for patients with mCRPC. While there is some evidence that pretreatment SUV_mean_ is a prognostic biomarker for poorer outcomes in mCRPC, the data from analyses of randomized controlled studies all provide compelling evidence that it may also have utility as a predictor of response to treatment with [^177^Lu]Lu-PSMA-617, given that higher whole-body tumor SUV_mean_ levels were consistently shown to predict favorable response to treatment in the TheraP and VISION trials (40, 43, 85). However, more data are needed, preferably from prospective randomized trials between treated and untreated comparator arms, regarding the utility of this biomarker as a predictor of response to [^177^Lu]Lu-PSMA-617. Likewise, stronger evidence for the potential of NLR and other hematologic biomarkers as predictive biomarkers, and for predicting hematotoxicity, is also required. Should future studies confirm their utility, NLR and hematologic biomarkers could see rapid uptake in clinical practice due to their relatively simple acquisition and interpretation. Nonetheless, clinically validated data are lacking with respect to biomarkers that can be utilized to predict response to [^177^Lu]Lu-PSMA-617 treatment. Although multivariate modeling of VISION data identified a number of parameters with potentially predictive value, these require evaluation in larger, biomarker-driven, randomized prospective trials. A summary of all the biomarkers discussed in this review is available in **Table 1**.

## 6 Conclusion

Although we provide insights into the potential role of certain biomarkers for providing prognostic and predictive utility, the field is still evolving and the biomarkers discussed need further research with appropriately designed trials before their value can be fully elucidated. It is also unlikely that any one biomarker will provide the adequate data needed for delivering optimal care to patients, so further work on producing nomograms or combinatorial biomarker scores will be required to help identify patients most suitable for treatment with [^177^Lu]Lu-PSMA-617. Improved understanding of the complex therapeutic environment that emerged with the approval of multiple life-prolonging agents will also be crucial to better integrate [^177^Lu]Lu-PSMA-617 in an effective treatment strategy. Ongoing discussions regarding how data from PSMA PET and other emergent biomarkers can become more widely used in clinical practice are therefore required. In the meantime, baseline whole-body PSMA PET visual assessments are possibly the most robust and readily available tool we currently have for prognosticating and predicting treatment response to [^177^Lu]Lu-PSMA-617, until obtaining whole-body SUV_mean_ data is more common in routine clinical practice.

## References

[1] National Cancer Institute. Cancer stat facts: prostate cancer (2022). Available online at: https://seer.cancer.gov/statfacts/html/prost.html (Accessed December, 2024).

[2] National Cancer Institute. Cancer stat facts: common cancer sites (2024). Available online at: https://seer.cancer.gov/statfacts/html/common.html (Accessed December, 2024).

[3] Poon DMC Cheung WSK Chiu PKF Chung DHS Kung JBT Lam DCM. Treatment of metastatic castration-resistant prostate cancer: review of current evidence and synthesis of expert opinions on radioligand therapy. Front Oncol. (2025) 15:1530580. doi: 10.3389/fonc.2025.1530580, PMID: 40071082 PMC11893367

[4] Bray F Laversanne M Sung H Ferlay J Siegel RL Soerjomataram I. Global cancer statistics 2022: GLOBOCAN estimates of incidence and mortality worldwide for 36 cancers in 185 countries. CA: A Cancer J Clin. (2024) 74:229–63. doi: 10.3322/caac.21834, PMID: 38572751

[5] Huang X Chau CH Figg WD. Challenges to improved therapeutics for metastatic castrate resistant prostate cancer: from recent successes and failures. J Hematol Oncol. (2012) 5:35. doi: 10.1186/1756-8722-5-35, PMID: 22747660 PMC3425086

[6] Le TK Duong QH Baylot V Fargette C Baboudjian M Colleaux L. Castration-resistant prostate cancer: from uncovered resistance mechanisms to current treatments. Cancers (Basel). (2023) 15. doi: 10.3390/cancers15205047, PMID: 37894414 PMC10605314

[7] Freedland SJ Davis M Epstein AJ Arondekar B Ivanova JI. Real-world treatment patterns and overall survival among men with Metastatic Castration-Resistant Prostate Cancer (mCRPC) in the US Medicare population. Prostate Cancer Prostatic Dis. (2024) 27:327–33. doi: 10.1038/s41391-023-00725-8, PMID: 37783836 PMC11096091

[8] Current K Meyer C Magyar CE Mona CE Almajano J Slavik R. Investigating PSMA-targeted radioligand therapy efficacy as a function of cellular PSMA levels and intratumoral PSMA heterogeneity. Clin Cancer Res. (2020) 26:2946–55. doi: 10.1158/1078-0432.Ccr-19-1485, PMID: 31932492 PMC7299755

[9] Yordanova A Eppard E Kürpig S Bundschuh RA Schönberger S Gonzalez-Carmona M. Theranostics in nuclear medicine practice. OncoTargets Ther. (2017) 10:4821–8. doi: 10.2147/ott.S140671, PMID: 29042793 PMC5633297

[10] Hope TA Aggarwal R Chee B Tao D Greene KL Cooperberg MR. Impact of (68)Ga-PSMA-11 PET on management in patients with biochemically recurrent prostate cancer. J Nucl Med. (2017) 58:1956–61. doi: 10.2967/jnumed.117.192476, PMID: 28522741

[11] Sartor AO Laidley D Pouliot F Probst S Sabbagh R Esposito G. A multicenter, randomized, controlled phase II study: Efficacy and safety of PSMA-targeted radioligand therapy I-131-1095 (1095) plus enzalutamide (enza) in 18F-DCFPyL PSMA scan avid, metastatic castration-resistant prostate cancer (mCRPC) patients post-abiraterone (abi) progression (ARROW). J Clin Oncol. (2021) 39:TPS187. doi: 10.1200/JCO.2021.39.6_suppl.TPS187

[12] Pomykala KL Czernin J Grogan TR Armstrong WR Williams J Calais J. Total-body (68)Ga-PSMA-11 PET/CT for bone metastasis detection in prostate cancer patients: potential impact on bone scan guidelines. J Nucl Med. (2020) 61:405–11. doi: 10.2967/jnumed.119.230318, PMID: 31541035 PMC7067527

[13] Bakht MK Beltran H. Biological determinants of PSMA expression, regulation and heterogeneity in prostate cancer. Nat Rev Urol. (2024) 22:26–45. doi: 10.1038/s41585-024-00900-z, PMID: 38977769 PMC11841200

[14] Al Saffar H Chen DC Delgado C Ingvar J Hofman MS Lawrentschuk N. The current landscape of prostate-specific membrane antigen (PSMA) imaging biomarkers for aggressive prostate cancer. Cancers (Basel). (2024) 16. doi: 10.3390/cancers16050939, PMID: 38473301 PMC10931387

[15] Ghosh A Heston WD. Tumor target prostate specific membrane antigen (PSMA) and its regulation in prostate cancer. J Cell Biochem. (2004) 91:528–39. doi: 10.1002/jcb.10661, PMID: 14755683

[16] Donin NM Reiter RE. Why targeting PSMA is a game changer in the management of prostate cancer. J Nucl Med. (2018) 59:177–82. doi: 10.2967/jnumed.117.191874, PMID: 28986509 PMC6910619

[17] European Medicines Agency. Pluvicto lutetium (177Lu) vipivotide tetraxetan (2022). Available online at: https://www.ema.europa.eu/en/medicines/human/EPAR/pluvicto (Accessed December, 2024).

[18] Novartis Pharmaceuticals Corporation. PLUVICTO^®^ (lutetium Lu 177 vipivotide tetraxetan) injection, for intravenous use (2025). Available online at: https://www.accessdata.fda.gov/drugsatfda_docs/label/2025/215833s021s024lbl.pdf (Accessed July, 2025).

[19] Sartor O de Bono J Chi KN Fizazi K Herrmann K Rahbar K. Lutetium-177-PSMA-617 for metastatic castration-resistant prostate cancer. N Engl J Med. (2021) 385:1091–103. doi: 10.1056/NEJMoa2107322, PMID: 34161051 PMC8446332

[20] Chi KN Metser U Czernin J Calais J Prasad V Eiber M. Study evaluating metastatic castrate resistant prostate cancer (mCRPC) treatment using 177Lu-PNT2002 PSMA therapy after second-line hormonal treatment (SPLASH). J Clin Oncol. (2021) 39. doi: 10.1200/JCO.2021.39.15_suppl.TPS5087

[21] Tagawa ST Vallabhajosula S Christos PJ Jhanwar YS Batra JS Lam L. Phase 1/2 study of fractionated dose lutetium-177-labeled anti-prostate-specific membrane antigen monoclonal antibody J591 ((177) Lu-J591) for metastatic castration-resistant prostate cancer. Cancer. (2019) 125:2561–9. doi: 10.1002/cncr.32072, PMID: 31012963 PMC13095068

[22] Johnson G Lengyelova E Nordquist LT Prasad V Anderson M Gervasio O. SECuRE: A dose escalation/expansion study to assess the anti-tumor efficacy of 67Cu-SAR-bisPSMA in patients with metastatic castrate-resistant prostate cancer. J Clin Oncol. (2024) 42:TPS246–TPS. doi: 10.1200/JCO.2024.42.4_suppl.TPS246

[23] Jang A Kendi AT Sartor O. Status of PSMA-targeted radioligand therapy in prostate cancer: current data and future trials. Ther Adv Med Oncol. (2023) 15:17588359231157632. doi: 10.1177/17588359231157632, PMID: 36895851 PMC9989419

[24] A phase I/​II, open-label, multi-center study of [225Ac]Ac-PSMA-R2 in men with PSMA-positive prostate cancer with or without prior 177Lu-psma radioligand therapy (2024). Available online at: https://clinicaltrials.gov/study/NCT05983198 (Accessed December, 2024).

[25] Sathekge M Bruchertseifer F Vorster M Lawal IO Knoesen O Mahapane J. mCRPC patients receiving (225)Ac-PSMA-617 therapy in the post-androgen deprivation therapy setting: response to treatment and survival analysis. J Nucl Med. (2022) 63:1496–502. doi: 10.2967/jnumed.121.263618, PMID: 35177427 PMC9536711

[26] Zacherl MJ Gildehaus FJ Mittlmeier L Böning G Gosewisch A Wenter V. First clinical results for PSMA-targeted α-therapy using (225)Ac-PSMA-I&T in advanced-mCRPC patients. J Nucl Med. (2021) 62:669–74. doi: 10.2967/jnumed.120.251017, PMID: 33008928

[27] Almeida LS Etchebehere E García Megías I Calapaquí Terán AK Hadaschik B Colletti PM. PSMA radioligand therapy in prostate cancer: where are we and where are we heading? Clin Nucl Med. (2024) 49:45–55. doi: 10.1097/rlu.0000000000004919, PMID: 37882758

[28] Wang H Remke M Horn T Schwamborn K Chen Y Steiger K. Heterogeneity of prostate-specific membrane antigen (PSMA) and PSMA-ligand uptake detection combining autoradiography and postoperative pathology in primary prostate cancer. EJNMMI Res. (2023) 13:99. doi: 10.1186/s13550-023-01044-8, PMID: 37971546 PMC10654338

[29] Pouliot F Saad F Rousseau E Richard PO Zamanian A Probst S. Intrapatient intermetastatic heterogeneity determined by triple-tracer PET imaging in mCRPC patients and correlation to survival: the 3TMPO cohort study. J Nucl Med. (2024) 65:1710–7. doi: 10.2967/jnumed.124.268020, PMID: 39327017 PMC11533914

[30] Hofman M Emmett L Sandhu S Iravani A Joshua A Goh J. ^177^Lu-PSMA-617 versus cabazitaxel in metastatic castration-resistant prostate cancer (TheraP): a randomised, open-label, phase 2 trial. Lancet. (2021) 397:797–804. doi: 10.1016/S0140-6736(21)00237-3, PMID: 33581798

[31] Bakht MK Yamada Y Ku SY Venkadakrishnan VB Korsen JA Kalidindi TM. Landscape of prostate-specific membrane antigen heterogeneity and regulation in AR-positive and AR-negative metastatic prostate cancer. Nat Cancer. (2023) 4:699–715. doi: 10.1038/s43018-023-00539-6, PMID: 37038004 PMC10867901

[32] FDA-NIH Biomarker Working Group. BEST (Biomarkers, endpointS, and other Tools) Resource [Internet]: Understanding Prognostic versus Predictive Biomarkers. Silver Spring (MD); Bethesda (MD: Food and Drug Administration (US); National Institutes of Health (US (2016). Available online at: https://www.ncbi.nlm.nih.gov/books/NBK402284/. 27010052

[33] Ballman KV. Biomarker: predictive or prognostic? J Clin Oncol. (2015) 33:3968–71. doi: 10.1200/jco.2015.63.3651, PMID: 26392104

[34] McShane LM Altman DG Sauerbrei W Taube SE Gion M Clark GM. Reporting recommendations for tumor marker prognostic studies (REMARK). J Natl Cancer Inst. (2005) 97:1180–4. doi: 10.1093/jnci/dji237, PMID: 16106022

[35] Şahin E Kefeli U Zorlu Ş Seyyar M Ozkorkmaz Akdag M Can Sanci P. Prognostic role of neutrophil-to-lymphocyte ratio, platelet-to-lymphocyte ratio, systemic immune-inflammation index, and pan-immune-inflammation value in metastatic castration-resistant prostate cancer patients who underwent 177Lu-PSMA-617. Med (Baltimore). (2023) 102:e35843. doi: 10.1097/md.0000000000035843, PMID: 38013293 PMC10681561

[36] Widjaja L Werner RA Ross TL Bengel FM Derlin T. Comparison of pretherapeutic osseous tumor volume and standard hematology for prediction of hematotoxicity after PSMA-targeted radioligand therapy. Eur J Nucl Med Mol Imaging. (2021) 48:4077–88. doi: 10.1007/s00259-021-05412-1, PMID: 34041564 PMC8484194

[37] Sartor O Ledet E Huang M Schwartz J Lieberman A Lewis B. Prediction of resistance to (177)Lu-PSMA therapy by assessment of baseline circulating tumor DNA biomarkers. J Nucl Med. (2023) 64:1721–5. doi: 10.2967/jnumed.123.266167, PMID: 37770113

[38] Tsechelidis I Vrachimis A. PSMA PET in imaging prostate cancer. Front Oncol. (2022) 12:831429. doi: 10.3389/fonc.2022.831429, PMID: 35155262 PMC8832487

[39] Kapoor M Kasi A. PET scanning. In: StatPearls. StatPearls Publishing, Treasure Island (FL (2024). 32644515

[40] Buteau JP Martin AJ Emmett L Iravani A Sandhu S Joshua AM. PSMA and FDG-PET as predictive and prognostic biomarkers in patients given [(177)Lu]Lu-PSMA-617 versus cabazitaxel for metastatic castration-resistant prostate cancer (TheraP): a biomarker analysis from a randomised, open-label, phase 2 trial. Lancet Oncol. (2022) 23:1389–97. doi: 10.1016/s1470-2045(22)00605-2 36261050

[41] Fendler WP Karpinski J Hüsing J Claassen K Möller L Kajüter H. Prognostic PSMA-PET PROMISE nomograms for patients with prostate cancer. J Clin Oncol. (2024) 42:5016. doi: 10.1200/JCO.2024.42.16_suppl.5016

[42] Hofman MS Emmett L Sandhu S Iravani A Buteau JP Joshua AM. Overall survival with [177Lu]Lu-PSMA-617 versus cabazitaxel in metastatic castration-resistant prostate cancer (TheraP): secondary outcomes of a randomised, open-label, phase 2 trial. Lancet Oncol. (2024) 25:99–107. doi: 10.1016/S1470-2045(23)00529-6 38043558

[43] Kuo PH Morris MJ Hesterman J Kendi AT Rahbar K Wei XX. Quantitative (68)Ga-PSMA-11 PET and clinical outcomes in metastatic castration-resistant prostate cancer following (177)Lu-PSMA-617 (VISION trial). Radiology. (2024) 312:e233460. doi: 10.1148/radiol.233460, PMID: 39162634 PMC11366674

[44] Berger A. How does it work? Positron emission tomography. BMJ. (2003) 326:1449. doi: 10.1136/bmj.326.7404.1449, PMID: 12829560 PMC1126321

[45] Wibmer AG Morris MJ Gonen M Zheng J Hricak H Larson S. Quantification of metastatic prostate cancer whole-body tumor burden with (18)F-FDG PET parameters and associations with overall survival after first-line abiraterone or enzalutamide: a single-center retrospective cohort study. J Nucl Med. (2021) 62:1050–6. doi: 10.2967/jnumed.120.256602, PMID: 33419944 PMC8833874

[46] Ferdinandus J Violet J Sandhu S Hicks RJ Ravi Kumar AS Iravani A. Prognostic biomarkers in men with metastatic castration-resistant prostate cancer receiving [177Lu]-PSMA-617. Eur J Nucl Med Mol Imaging. (2020) 47:2322. doi: 10.1007/s00259-020-04723-z, PMID: 32140802

[47] Im HJ Bradshaw T Solaiyappan M Cho SY. Current methods to define metabolic tumor volume in positron emission tomography: which one is better? Nucl Med Mol Imaging. (2018) 52:5–15. doi: 10.1007/s13139-017-0493-6, PMID: 29391907 PMC5777960

[48] Kuo PH Benson T Messmann R Groaning M. Why we did what we did: PSMA PET/CT selection criteria for the VISION Trial. J Nucl Med. (2022) 63:816–8. doi: 10.2967/jnumed.121.263638, PMID: 35086895

[49] Buck AK Nekolla S Ziegler S Beer A Krause BJ Herrmann K. SPECT/CT. J Nucl Med. (2008) 49:1305–19. doi: 10.2967/jnumed.107.050195, PMID: 18632825

[50] Pathmanandavel S Crumbaker M Ho B Yam AO Wilson P Niman R. Evaluation of (177)Lu-PSMA-617 SPECT/CT quantitation as a response biomarker within a prospective (177)Lu-PSMA-617 and NOX66 combination trial (LuPIN). J Nucl Med. (2023) 64:221–6. doi: 10.2967/jnumed.122.264398, PMID: 36008120 PMC9902857

[51] Neubauer MC Nicolas GP Bauman A Fani M Nitzsche E Afshar-Oromieh A. Early response monitoring during [(177)Lu]Lu-PSMA I&T therapy with quantitated SPECT/CT predicts overall survival of mCRPC patients: subgroup analysis of a Swiss-wide prospective registry study. Eur J Nucl Med Mol Imaging. (2024) 51:1185–93. doi: 10.1007/s00259-023-06536-2, PMID: 38038755 PMC10881597

[52] Demirci RA Gulati R Hawley JE Yezefski T Haffner MC Cheng HH. SPECT/CT in early response assessment of patients with metastatic castration-resistant prostate cancer receiving (177)Lu-PSMA-617. J Nucl Med. (2024) 65:1945–51. doi: 10.2967/jnumed.124.267665, PMID: 39510589 PMC11937724

[53] Emmett L John N Pathmanandavel S Counter W Ayers M Sharma S. Patient outcomes following a response biomarker-guided approach to treatment using 177Lu-PSMA-I&T in men with metastatic castrate-resistant prostate cancer (Re-SPECT). Ther Adv Med Oncol. (2023) 15:17588359231156392. doi: 10.1177/17588359231156392, PMID: 36872949 PMC9983078

[54] John N Pathmanandavel S Crumbaker M Counter W Ho B Yam AO. (177)Lu-PSMA SPECT quantitation at 6 weeks (dose 2) predicts short progression free survival for patients undergoing lu PSMA I&T therapy. J Nucl Med. (2022) 64:410–5. doi: 10.2967/jnumed.122.264677, PMID: 36215568

[55] Yadav S Lowery B Tuchayi AM Jiang F Saelee R Aggarwal RR. Impact of posttreatment SPECT/CT on patient management during (177)Lu-PSMA-617 radiopharmaceutical therapy. J Nucl Med. (2024) 65:1395–401. doi: 10.2967/jnumed.124.267955, PMID: 39117452 PMC13671392

[56] Yadav S Jiang F Kurkowska S Saelee R Morley A Feng F. Assessing response to PSMA radiopharmaceutical therapies with single SPECT imaging at 24 hours after injection. J Nucl Med. (2024) 65:1064–9. doi: 10.2967/jnumed.123.267208, PMID: 38724282 PMC13671390

[57] Song H Ferri V Duan H Aparici CM Davidzon G Franc BL. SPECT at the speed of PET: a feasibility study of CZT-based whole-body SPECT/CT in the post (177)Lu-DOTATATE and (177)Lu-PSMA617 setting. Eur J Nucl Med Mol Imaging. (2023) 50:2250–7. doi: 10.1007/s00259-023-06176-6, PMID: 36869177

[58] Swiha M Pathmanandavel S Papa N Sabahi Z Li S Zheng A. Comparison of posttherapy 4- and 24-hour [(177)Lu]Lu-PSMA SPECT/CT and pretherapy PSMA PET/CT in assessment of disease in men with metastatic castration-resistant prostate cancer. J Nucl Med. (2024) 65:1939–44. doi: 10.2967/jnumed.124.267606, PMID: 39477497

[59] Waninger JJ Ma VT Chopra Z Pearson AN Green MD. Evaluation of the prognostic role of liver metastases on patient outcomes: systematic review and meta-analysis. Cancer J. (2023) 29:279–84. doi: 10.1097/ppo.0000000000000683, PMID: 37796646 PMC10558088

[60] Halabi S Kelly WK Ma H Zhou H Solomon NC Fizazi K. Meta-analysis evaluating the impact of site of metastasis on overall survival in men with castration-resistant prostate cancer. J Clin Oncol. (2016) 34:1652–9. doi: 10.1200/JCO.2015.65.7270, PMID: 26951312 PMC4872320

[61] Ahmadzadehfar H Rahbar K Baum RP Seifert R Kessel K Bögemann M. Prior therapies as prognostic factors of overall survival in metastatic castration-resistant prostate cancer patients treated with [(177)Lu]Lu-PSMA-617. A WARMTH multicenter study (the 617 trial). Eur J Nucl Med Mol Imaging. (2021) 48:113–22. doi: 10.1007/s00259-020-04797-9, PMID: 32383093 PMC7835179

[62] Rahbar K Ahmadzadehfar H Kratochwil C Haberkorn U Schäfers M Essler M. German multicenter study investigating 177Lu-PSMA-617 radioligand therapy in advanced prostate cancer patients. J Nucl Med. (2017) 58:85–90. doi: 10.2967/jnumed.116.183194, PMID: 27765862

[63] Wrenger R Jüptner M Marx M Zhao Y Zuhayra M Caliebe A. Pre- and intratherapeutic predictors of overall survival in patients with advanced metastasized castration-resistant prostate cancer receiving Lu-177-PSMA-617 radioligand therapy. BMC Urol. (2022) 22:96. doi: 10.1186/s12894-022-01050-3, PMID: 35788220 PMC9254582

[64] Telli T Tuncel M Karabulut E Aksoy S Erman M Akdogan B. Prognostic factors of overall and prostate-specific antigen-progression-free survival in metastatic castration-resistant prostate cancer patients treated with (177) Lu-PSMA-617. A single-center prospective observational study. Prostate. (2023) 83:792–800. doi: 10.1002/pros.24518, PMID: 36919876

[65] Derlin T Riethdorf S Schumacher U Lafos M Peine S Coith C. PSMA-heterogeneity in metastatic castration-resistant prostate cancer: circulating tumor cells, metastatic tumor burden, and response to targeted radioligand therapy. Prostate. (2023) 83:1076–88. doi: 10.1002/pros.24549, PMID: 37147881

[66] Fonseca NM Maurice-Dror C Herberts C Tu W Fan W Murtha AJ. Prediction of plasma ctDNA fraction and prognostic implications of liquid biopsy in advanced prostate cancer. Nat Commun. (2024) 15:1828. doi: 10.1038/s41467-024-45475-w, PMID: 38418825 PMC10902374

[67] Reichert ZR Morgan TM Li G Castellanos E Snow T Dall’Olio FG. Prognostic value of plasma circulating tumor DNA fraction across four common cancer types: a real-world outcomes study. Ann Oncol. (2023) 34:111–20. doi: 10.1016/j.annonc.2022.09.163, PMID: 36208697 PMC9805517

[68] Bono JSD Morris MJ Sartor O Wei XX Fizazi K Herrmann K. Baseline ctDNA analyses and associations with outcomes in taxane-naive patients with mCRPC treated with 177Lu-PSMA-617 versus change of ARPI in PSMAfore. J Clin Oncol. (2024) 42:5008. doi: 10.1200/JCO.2024.42.16_suppl.5008

[69] Vanwelkenhuyzen J Van Bos E Van Bruwaene S Lesage K Maes A Üstmert S. AR and PI3K genomic profiling of cell-free DNA can identify poor responders to Lutetium-177-PSMA among patients with metastatic castration-resistant prostate cancer. Eur Urol Open Sci. (2023) 53:63–6. doi: 10.1016/j.euros.2023.05.008, PMID: 37292496 PMC10244905

[70] Kwan EM Ng SWS Tolmeijer SH Emmett L Sandhu S Buteau JP. Lutetium-177-PSMA-617 or cabazitaxel in metastatic prostate cancer: circulating tumor DNA analysis of the randomized phase 2 TheraP trial. Nature medicine. doi: 10.1038/s41591-025-03704-9, PMID: 40425844

[71] NCCN Clinical Practice Guidelines in Oncology (NCCN Guidelines®) for Prostate Cancer V.1.2025. © National Comprehensive Cancer Network, Inc. (2025). All rights reserved. Available online at: https://www.nccn.org/ (Accessed June 2025).

[72] Raychaudhuri R Mo G Haffner MC Morrissey C Ha G Yu EY. PSMA expression and response to 177Lu-PSMA-617 (LuPSMA) in men with vs. without DNA damage repair (DDR) mutations. J Clin Oncol. (2023) 41:5055. doi: 10.1200/JCO.2023.41.16_suppl.5055

[73] Crumbaker M Goldstein LD Murray DH Tao J Pathmanandavel S Boulter N. Circulating tumour DNA biomarkers associated with outcomes in metastatic prostate cancer treated with Lutetium-177-PSMA-617. Eur Urol Open Sci. (2023) 57:30–6. doi: 10.1016/j.euros.2023.08.007, PMID: 38020530 PMC10658415

[74] Panian J Henderson N Barata PC Bilen MA Graham L Heath E. Association of tumor genetics with outcomes in patients (pts) with PSMA-positive metastatic castration-resistant prostate cancer (mCRPC) treated with 177Lu-PSMA-617. J Clin Oncol. (2024) 42. doi: 10.1200/JCO.2024.42.16_suppl.5050 PMC12622373 41124032

[75] Gauntner T Gheeya JS Khorasanchi A Xu M Hasanov E Collier KA. Association of genomic alterations with clinical outcomes following lutetium-177-PSMA vipivotide tetraxetan in men with metastatic castrate-resistant prostate cancer. J Clin Oncol. (2024) 42. doi: 10.1200/JCO.2024.42.16_suppl.5057

[76] Yilmaz B Chalker C Yu Y Kaempf A Mittra E MallaK N. Genetic analysis of metastatic castration resistant prostate cancer patients treated with Lu-177 PSMA radioligand therapy. J Clin Oncol. (2024) 42:e17063. doi: 10.1200/JCO.2024.42.16_suppl.e17063

[77] Rajwa P Zapała P Merseburger AS. Targeting androgen receptor alterations in metastatic prostate cancer. Eur Urol Focus. (2024) 11:79–81. doi: 10.1016/j.euf.2024.07.012, PMID: 39107195

[78] Quigley DA Dang HX Zhao SG Lloyd P Aggarwal R Alumkal JJ. Genomic hallmarks and structural variation in metastatic prostate cancer. Cell. (2018) 174:758–69.e9. doi: 10.1016/j.cell.2018.06.039, PMID: 30033370 PMC6425931

[79] Jernberg E Bergh A Wikström P. Clinical relevance of androgen receptor alterations in prostate cancer. Endocr Connect. (2017) 6:R146–R61. doi: 10.1530/ec-17-0118, PMID: 29030409 PMC5640574

[80] De Giorgi U Sansovini M Severi S Nicolini S Monti M Gurioli G. Circulating androgen receptor gene amplification and resistance to (177)Lu-PSMA-617 in metastatic castration-resistant prostate cancer: results of a Phase 2 trial. Br J Cancer. (2021) 125:1226–32. doi: 10.1038/s41416-021-01508-5, PMID: 34333554 PMC8548293

[81] Kessel K Seifert R Weckesser M Roll W Humberg V Schlack K. Molecular analysis of circulating tumor cells of metastatic castration-resistant prostate cancer patients receiving (177)Lu-PSMA-617 radioligand therapy. Theranostics. (2020) 10:7645–55. doi: 10.7150/thno.44556, PMID: 32685010 PMC7359074

[82] Stangl-Kremser J Sun M Ho B Thomas J Nauseef JT Osborne JR. Prognostic value of neutrophil-to-lymphocyte ratio in patients with metastatic castration-resistant prostate cancer receiving prostate-specific membrane antigen targeted radionuclide therapy. Prostate. (2023) 83:1351–7. doi: 10.1002/pros.24597, PMID: 37424145

[83] Cupp MA Cariolou M Tzoulaki I Aune D Evangelou E Berlanga-Taylor AJ. Neutrophil to lymphocyte ratio and cancer prognosis: an umbrella review of systematic reviews and meta-analyses of observational studies. BMC Med. (2020) 18:360. doi: 10.1186/s12916-020-01817-1, PMID: 33213430 PMC7678319

[84] Hermann K Gafita A de Bono J Sartor AO Chi KN Krause BJ. Building a predictive model for outcomes with [177Lu]Lu-PSMA-617 in patients with metastatic castration-resistant prostate cancer using VISION data. J Clin Oncol. (2024) 42:208. doi: 10.1200/JCO.2024.42.4_suppl.208

[85] Kuo P Hesterman J Rahbar K Kendi AT Wei XX Fang B. 68Ga]Ga-PSMA-11 PET baseline imaging as a prognostic tool for clinical outcomes to [177Lu]Lu-PSMA-617 in patients with mCRPC: A VISION substudy. J Clin Oncol. (2022) 40:5002. doi: 10.1200/JCO.2022.40.16_suppl.5002

[86] Kuo PH Morris M Kendi AT Rahbar K Wei XX Fang B. Association of baseline quantitative [68Ga]Ga-PSMA-11 PET imaging parameters with clinical outcomes in patients with mCRPC receiving [177Lu]Lu-PSMA-617: a VISION sub-study. Eur J Nucl Med Mol Imaging. (2023) 50:S155. doi: 10.1007/s00259-023-06333-x, PMID: 37594496

[87] Swiha M Papa N Sabahi Z Ayati N John N Pathmanandavel S. Development of a visually calculated SUV(mean) (HIT score) on screening PSMA PET/CT to predict treatment response to (177)Lu-PSMA therapy: comparison with quantitative SUV(mean) and patient outcomes. J Nucl Med. (2024) 65:904–8. doi: 10.2967/jnumed.123.267014, PMID: 38637137

[88] FDA Press Release. FDA approves first PSMA-targeted PET imaging drug for men with prostate cancer (2020). Available online at: https://www.fda.gov/news-events/press-announcements/fda-approves-first-psma-targeted-pet-imaging-drug-men-prostate-cancer (Accessed December, 2024).

[89] Garje R Hope TA Rumble RB Parikh RA. Systemic therapy update on (177)Lutetium-PSMA-617 for metastatic castration-resistant prostate cancer: ASCO guideline rapid recommendation Q and A. JCO Oncol Pract. (2023) 19:132–5. doi: 10.1200/op.22.00753, PMID: 36693228

[90] Maslov DV New J Patel N Kosty MO Bhangoo MS. Pluvicto in the real world setting and timing to PSA50 as a predictive marker of treatment response. J Clin Oncol. (2024) 42:e17026. doi: 10.1200/JCO.2024.42.16_suppl.e17026

[91] Balachandran VP Gonen M Smith JJ DeMatteo RP. Nomograms in oncology: more than meets the eye. Lancet Oncol. (2015) 16:e173–80. doi: 10.1016/s1470-2045(14)71116-7, PMID: 25846097 PMC4465353

[92] Karpinski MJ Hüsing J Claassen K Möller L Kajüter H Oesterling F. Combining PSMA-PET and PROMISE to re-define disease stage and risk in patients with prostate cancer: a multicentre retrospective study. Lancet Oncol. (2024) 25:1188–201. doi: 10.1016/s1470-2045(24)00326-7, PMID: 39089299

[93] Gafita A Calais J Grogan TR Hadaschik B Wang H Weber M. Nomograms to predict outcomes after (177)Lu-PSMA therapy in men with metastatic castration-resistant prostate cancer: an international, multicentre, retrospective study. Lancet Oncol. (2021) 22:1115–25. doi: 10.1016/S1470-2045(21)00274-6, PMID: 34246328

[94] Sauerbrei W Haeussler T Balmford J Huebner M. Structured reporting to improve transparency of analyses in prognostic marker studies. BMC Med. (2022) 20:184. doi: 10.1186/s12916-022-02304-5, PMID: 35546237 PMC9095054

[95] Azuaje F Devaux Y Wagner D. Challenges and standards in reporting diagnostic and prognostic biomarker studies. Clin Transl Sci. (2009) 2:156–61. doi: 10.1111/j.1752-8062.2008.00075.x, PMID: 20443882 PMC5350675

[96] Kyzas PA Loizou KT Ioannidis JP. Selective reporting biases in cancer prognostic factor studies. J Natl Cancer Inst. (2005) 97:1043–55. doi: 10.1093/jnci/dji184, PMID: 16030302

[97] Sheehan B Guo C Neeb A Paschalis A Sandhu S de Bono JS. Prostate-specific membrane antigen biology in lethal prostate cancer and its therapeutic implications. Eur Urol Focus. (2022) 8:1157–68. doi: 10.1016/j.euf.2021.06.006, PMID: 34167925

[98] Hyväkkä A Virtanen V Kemppainen J Grönroos TJ Minn H Sundvall M. More than meets the eye: Scientific rationale behind molecular imaging and therapeutic targeting of prostate-specific membrane antigen (PSMA) in metastatic prostate cancer and beyond. Cancers (Basel). (2021) 13:2244. doi: 10.3390/cancers13092244, PMID: 34067046 PMC8125679

[99] Lückerath K Wei L Fendler WP Evans-Axelsson S Stuparu AD Slavik R. Preclinical evaluation of PSMA expression in response to androgen receptor blockade for theranostics in prostate cancer. EJNMMI Res. (2018) 8:96. doi: 10.1186/s13550-018-0451-z, PMID: 30374743 PMC6206308

[100] Sommer U Siciliano T Ebersbach C Beier AK Stope MB Jöhrens K. Impact of androgen receptor activity on prostate-specific membrane antigen expression in prostate cancer cells. Int J Mol Sci. (2022) 23:1046. doi: 10.3390/ijms23031046, PMID: 35162969 PMC8835452

[101] Kessel K Bernemann C Bögemann M Rahbar K. Evolving castration resistance and prostate specific membrane antigen expression: implications for patient management. Cancers (Basel). (2021) 13:3556. doi: 10.3390/cancers13143556, PMID: 34298770 PMC8307676

